# Supplementary material for: Cell-intrinsic regulation of phagocyte function by interferon lambda during pulmonary viral, bacterial super-infection
Source: PLoS Pathog. 2024 Aug 23;20(8):e1012498. doi: 10.1371/journal.ppat.1012498 (PMC11376568; doi:10.1371/journal.ppat.1012498)
Supplement: S1 Fig — A. Super-infected global IFNLR1-/- mice show trending reductions in bacterial burden 6 hours post bacterial infection compared to WT mice (IFNLR1-/- n = 6, WT n = 6, data from 2 replicates). B. Protein levels in the airways of super-infected mice were evaluated 6 hours post super-infection onset (IFNLR1-/- n = 10, WT n = 10). C. Mouse weight loss was monitored daily during infection time-course (data from 2 replicates). D. Immune cell infiltration into the airways was assessed by quantification of cells in the BAL fluid 6 hours post super-infection (IFNLR1-/- n = 4, WT n = 4, representative data from 2 replicates). E. Global cytokine levels were detected using Bio-Plex assays in super-infected mice 6 hours post bacterial infection (IFNLR1-/- n = 10, WT n = 10). p values: *<0.05, **<0.01, ***<0.001, ****<0.0001. (PDF) [file ppat.1012498.s001.pdf]

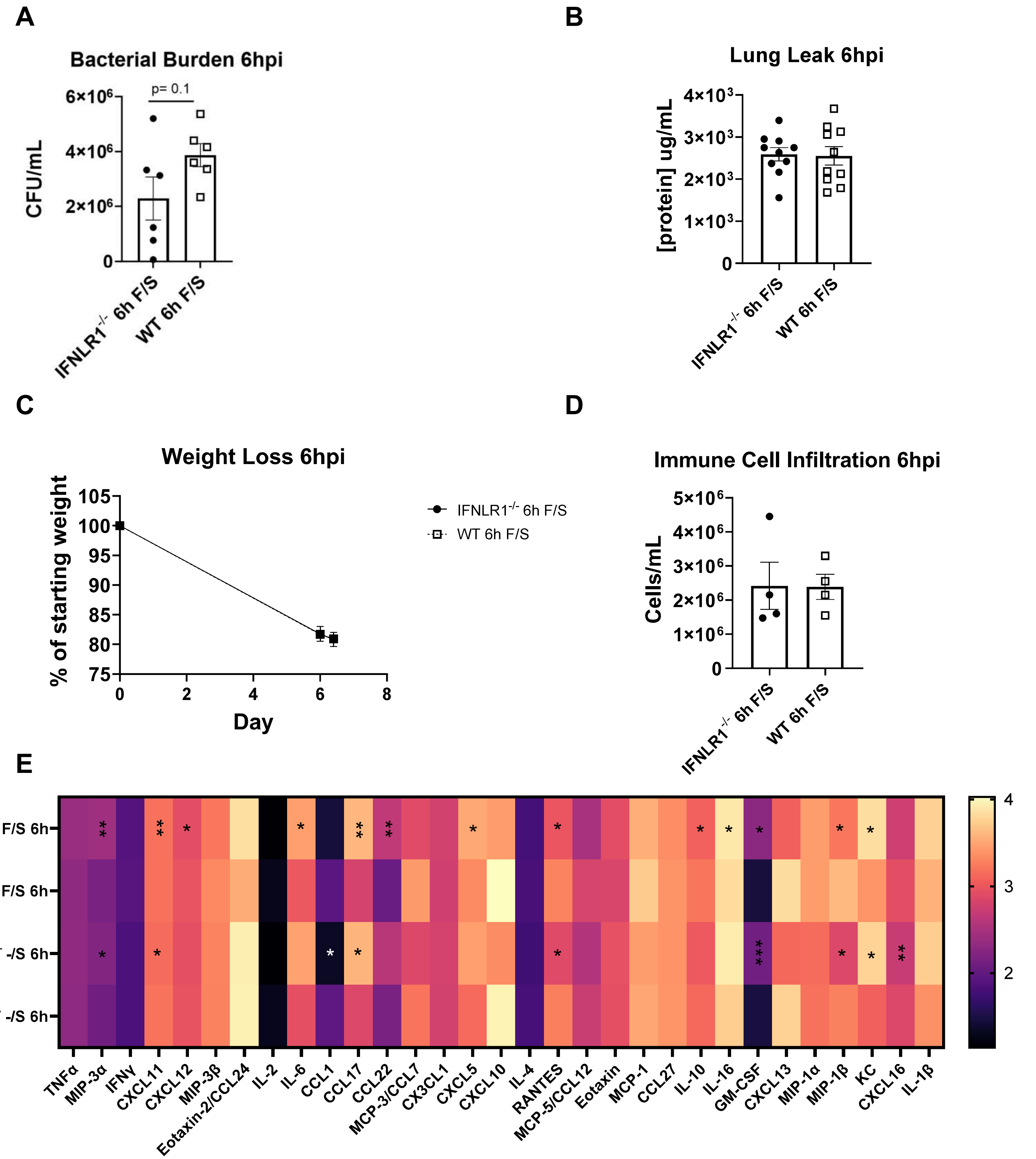

S1 Figure. Global IFNLR1$^{-/-}$ mice are comparable to WT at early super-infection timepoints. A. Super-infected global IFNLR1$^{-/-}$ mice show trending reductions in bacterial burden 6 hours post bacterial infection compared to WT mice (IFNLR1$^{-/-}$ n=6, WT n=6, data from 2 replicates). B. Protein levels in the airways of super-infected mice were evaluated 6 hours post super-infection onset (IFNLR1$^{-/-}$ n=10, WT n=10). C. Mouse weight loss was monitored daily during infection time-course (data from 2 replicates). D. Immune cell infiltration into the airways was assessed by quantification of cells in the BAL fluid 6 hours post super-infection (IFNLR1$^{-/-}$ n=4, WT n=4, representative data from 2 replicates). E. Global cytokine levels were detected using Bio-Plex assays in super-infected mice 6 hours post bacterial infection (IFNLR1$^{-/-}$ n=10, WT n=10). p values: *<0.05, **<0.01, ***<0.001, ****<0.0001
