# Supplementary material for: Cell-intrinsic regulation of phagocyte function by interferon lambda during pulmonary viral, bacterial super-infection
Source: PLoS Pathog. 2024 Aug 23;20(8):e1012498. doi: 10.1371/journal.ppat.1012498 (PMC11376568; doi:10.1371/journal.ppat.1012498)
Supplement: S2 Fig — A. Pathology scores were determined by Qupath software analysis of perivascular, peribronchial, and parenchymal lung damage (IFNLR1-/- n = 11, WT n = 12). B. Representative images of whole lung slides after H&E staining (IFNLR1-/- n = 2, WT n = 2). C. Levels of ifnlr1 transcript were assessed in WT and IFNLR1-/- CD11c+ cells by qPCR. Cells were sorted from naïve mice (n = 4). p values: *<0.05, **<0.01, ***<0.001, ****<0.0001. (PDF) [file ppat.1012498.s002.pdf]

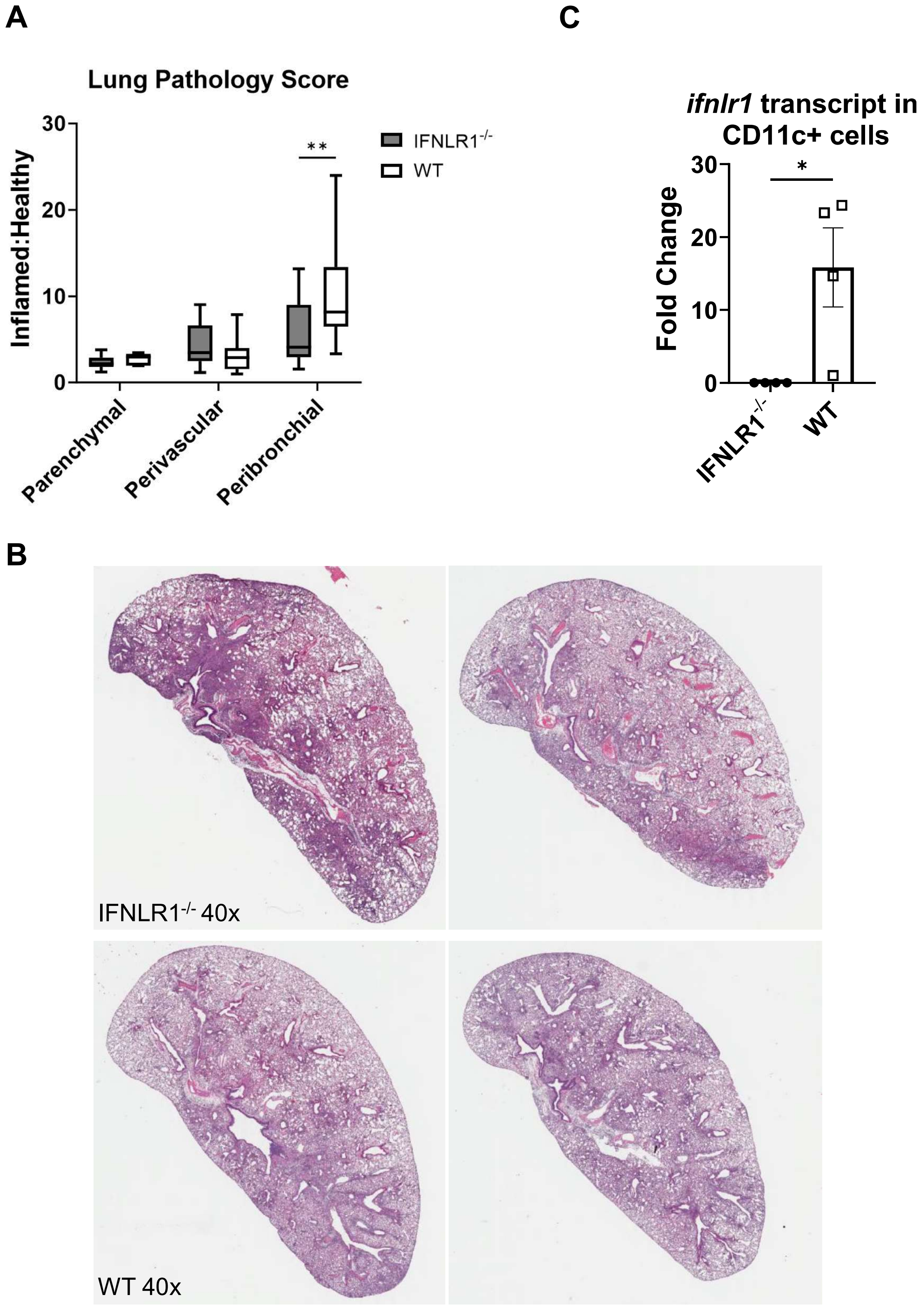

S2 Figure. Global IFNLR1$^{-/-}$ alters immunopathology after super-infection. A. Pathology scores were determined by Qupath software analysis of perivascular, peribronchial, and parenchymal lung damage (IFNLR1$^{-/-}$ n=11, WT n=12). B. Representative images of whole lung slides after H&E staining (IFNLR1$^{-/-}$ n=2, WT n=2). C. Levels of *ifnlr1* transcript were assessed in WT and IFNLR1$^{-/-}$ CD11c+ cells by qPCR. Cells were sorted from naïve mice (n=4). p values: *<0.05, **<0.01, ***<0.001, ****<0.0001
