# Supplementary material for: Cell-intrinsic regulation of phagocyte function by interferon lambda during pulmonary viral, bacterial super-infection
Source: PLoS Pathog. 2024 Aug 23;20(8):e1012498. doi: 10.1371/journal.ppat.1012498 (PMC11376568; doi:10.1371/journal.ppat.1012498)
Supplement: S3 Fig — A. Representative flow plots of non-phagocytic CD90+ cells show gating strategy for dsRed+ cells in WT (left) and IFNLR1-/- mice (right). B. Frequency (left) and total cell counts (right) of dsRed+ CD11b+ dendritic cells (DCs; CD45+, CD11c+, CD11b+, CD103-) are increased in IFNLR1-/- mice compared to WT during super-infection (representative data, n = 4 for all groups). C. Total cell counts of dsRed+ inflammatory monocytes, lung monocytes, interstitial macrophages, and neutrophils largely recapitulate frequencies of dsRed+ cells seen in Fig 2A (representative data, n = 4 for all groups). D. Overall immune cell recruitment measured by flow cytometry showed no differences in cell count (top) or frequency (bottom) in super-infected IFNLR1-/- versus WT mice (top: IFNLR1-/- n = 8, WT n = 8; bottom: IFNLR1-/- n = 14, WT n = 14). p values: *<0.05, **<0.01, ***<0.001, ****<0.0001. (PDF) [file ppat.1012498.s003.pdf]

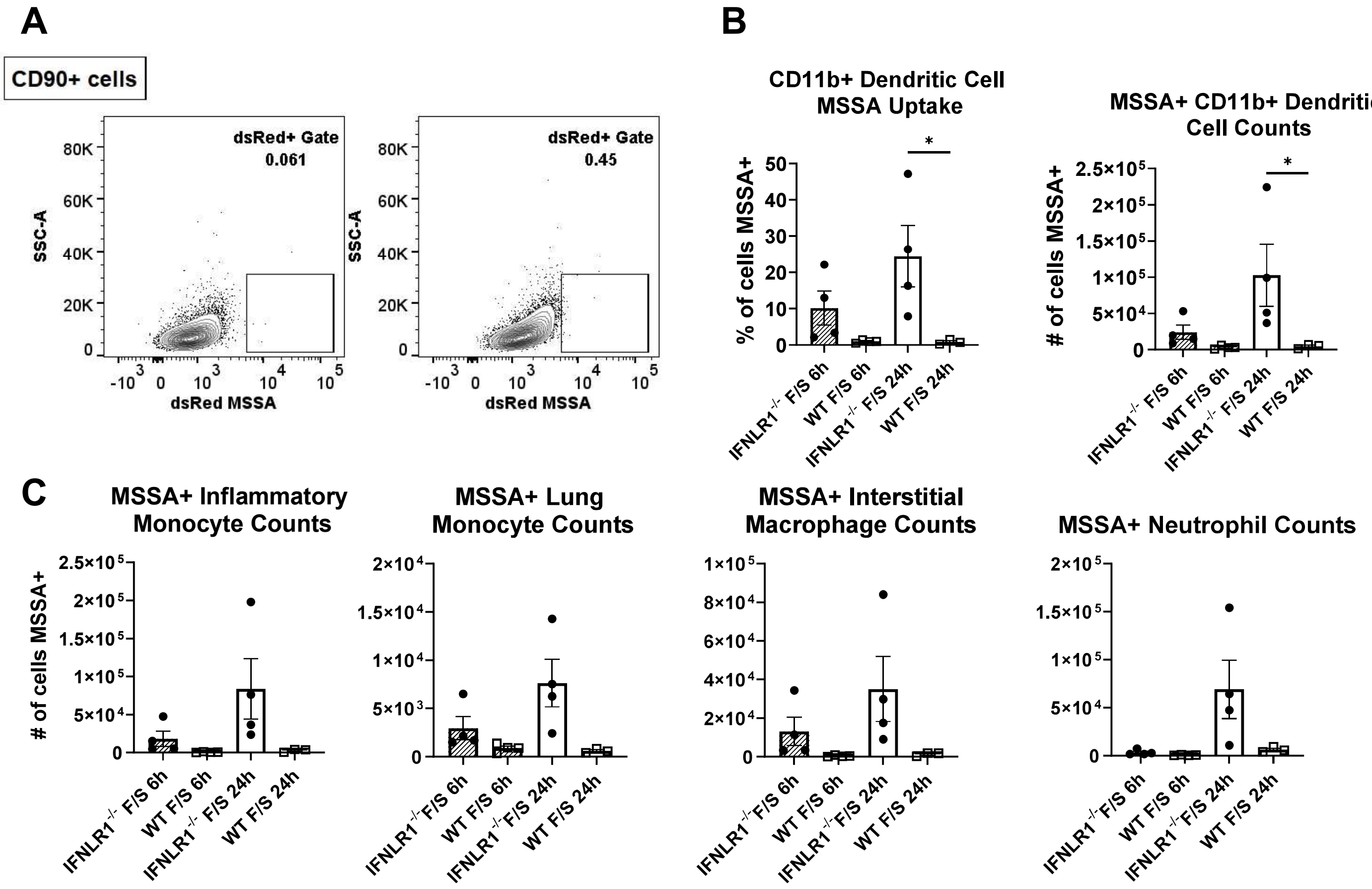

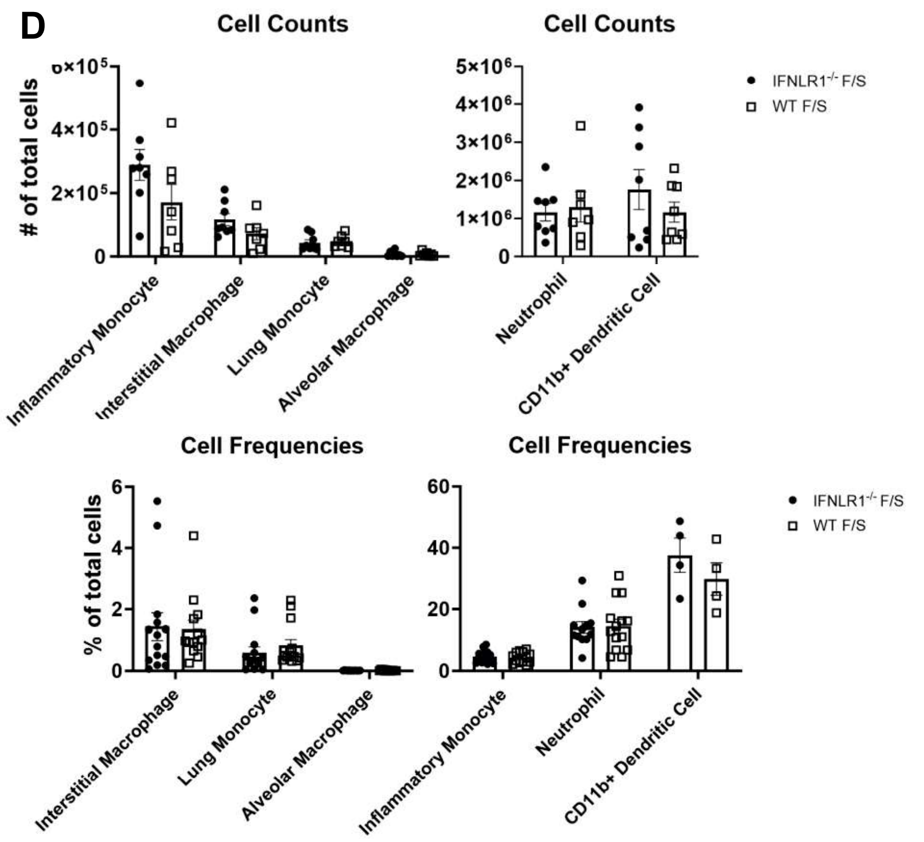

S3 Figure. IFNλ disrupts phagocytosis of multiple phagocyte populations during super-infection. A. Representative flow plots of non-phagocytic CD90+ cells show gating strategy for dsRed+ cells in WT (left) and IFNLR1$^{-/-}$ mice (right). B. Frequency (left) and total cell counts (right) of dsRed+ CD11b+ dendritic cells (DCs; CD45^{+}, CD11c^{+}, CD11b^{+}, CD103^{-}) are increased in IFNLR1$^{-/-}$ mice compared to WT during super-infection (representative data, n=4 for all groups). C. Total cell counts of dsRed+ inflammatory monocytes, lung monocytes, interstitial macrophages, and neutrophils largely recapitulate frequencies of dsRed+ cells seen in Figure 3A (representative data, n=4 for all groups). D. Overall immune cell recruitment measured by flow cytometry showed no differences in cell count (top) or frequency (bottom) in super-infected IFNLR1$^{-/-}$ versus WT mice (top: IFNLR1$^{-/-}$ n=8, WT n=8; bottom: IFNLR1$^{-/-}$ n=14, WT n=14). p values: *<0.05, **<0.01, ***<0.001, ****<0.0001
