# Supplementary material for: Cell-intrinsic regulation of phagocyte function by interferon lambda during pulmonary viral, bacterial super-infection
Source: PLoS Pathog. 2024 Aug 23;20(8):e1012498. doi: 10.1371/journal.ppat.1012498 (PMC11376568; doi:10.1371/journal.ppat.1012498)
Supplement: S4 Fig — A. Frequencies of pHrodo red+ cells in global IFNLR1-/- and WT mice during super-infection (n = 6). B. Representative flow plots from super-infected global IFNLR1-/- (top) and WT (bottom) mice. Mice were infected with 900 PFU PR8 and 5 x 10^7^ CFU pHrodo-red labeled MRSA strain USA300. Global IFNLR1-/- mice have increased uptake of bacteria as quantified by pHrodo red+ cells compared to WT mice, consistent with Fig 3A. (PDF) [file ppat.1012498.s004.pdf]

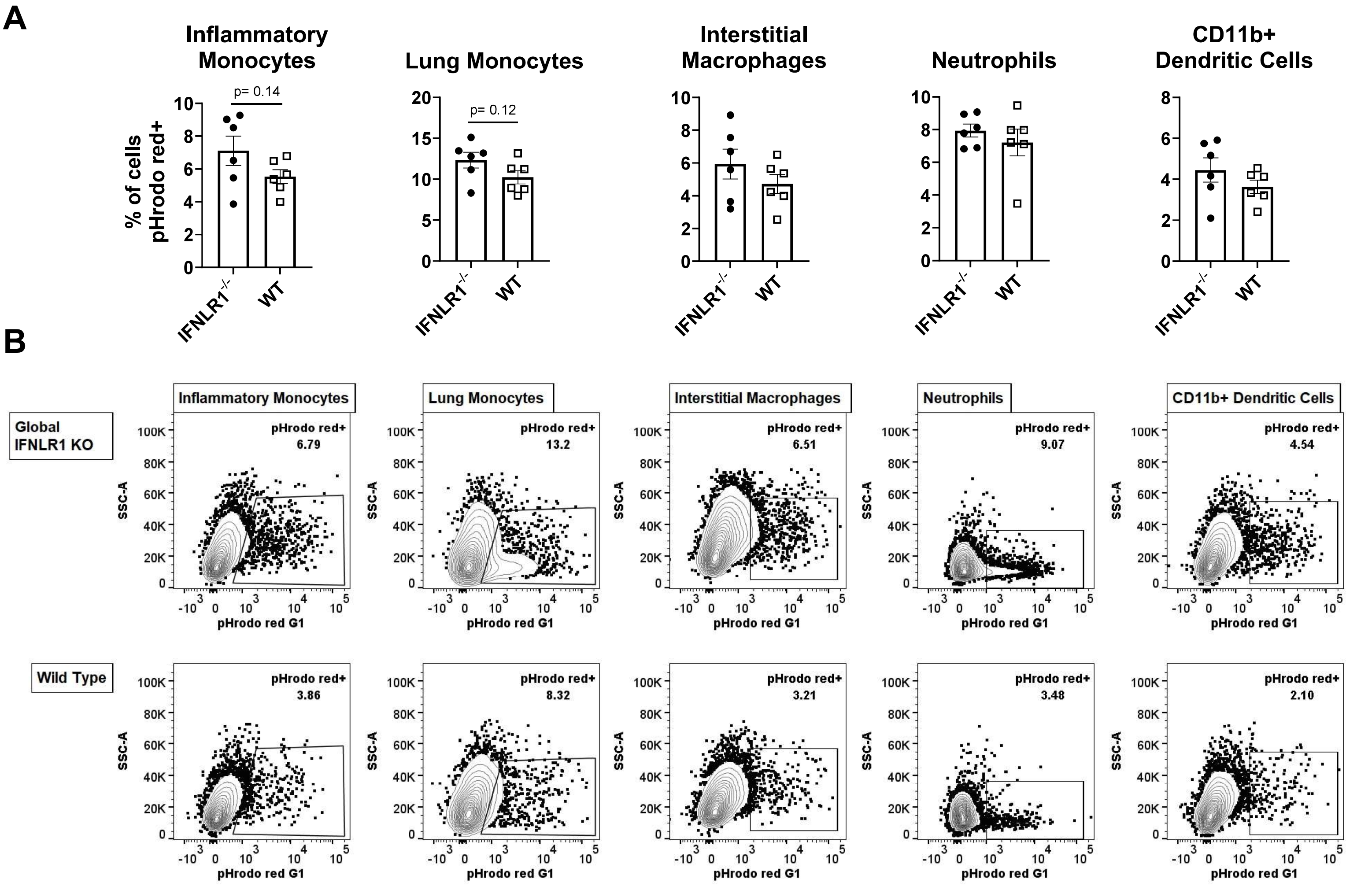

S4 Figure. IFNλ disrupts phagocytosis of multiple phagocyte populations during super-infection. A. Frequencies of pHrodo red+ cells in global IFNLR1$^{-/-}$ and WT mice during super-infection (n=6). B. Representative flow plots from super-infected global IFNLR1$^{-/-}$ (top) and WT (bottom) mice. Mice were infected with 900 PFU PR8 and 5 x 10^7 CFU pHrodo-red labeled MRSA strain USA300. Global IFNLR1$^{-/-}$ mice have increased uptake of bacteria as quantified by pHrodo red+ cells compared to WT mice, consistent with Fig 3A.
