# Supplementary material for: Cell-intrinsic regulation of phagocyte function by interferon lambda during pulmonary viral, bacterial super-infection
Source: PLoS Pathog. 2024 Aug 23;20(8):e1012498. doi: 10.1371/journal.ppat.1012498 (PMC11376568; doi:10.1371/journal.ppat.1012498)
Supplement: S5 Fig — A. WT or IFNLR1-/- mice were sublethally irradiated and reconstituted with 1:1 WT:IFNLR1-/- bone marrow for 9 weeks before being super-infected. Figure created on Biorender.com. B. Reconstitution of bone marrow cells was comparable between WT and IFNLR1-/- cells in both mouse backgrounds. C. Total counts of immune cell recruitment to the lung was not significantly altered by host genotype or immune cell genotype, with the exception of neutrophils in IFNLR1-/- hosts (host: KO n = 8, WT n = 8). p values: *<0.05, **<0.01, ***<0.001, ****<0.0001. (PDF) [file ppat.1012498.s005.pdf]

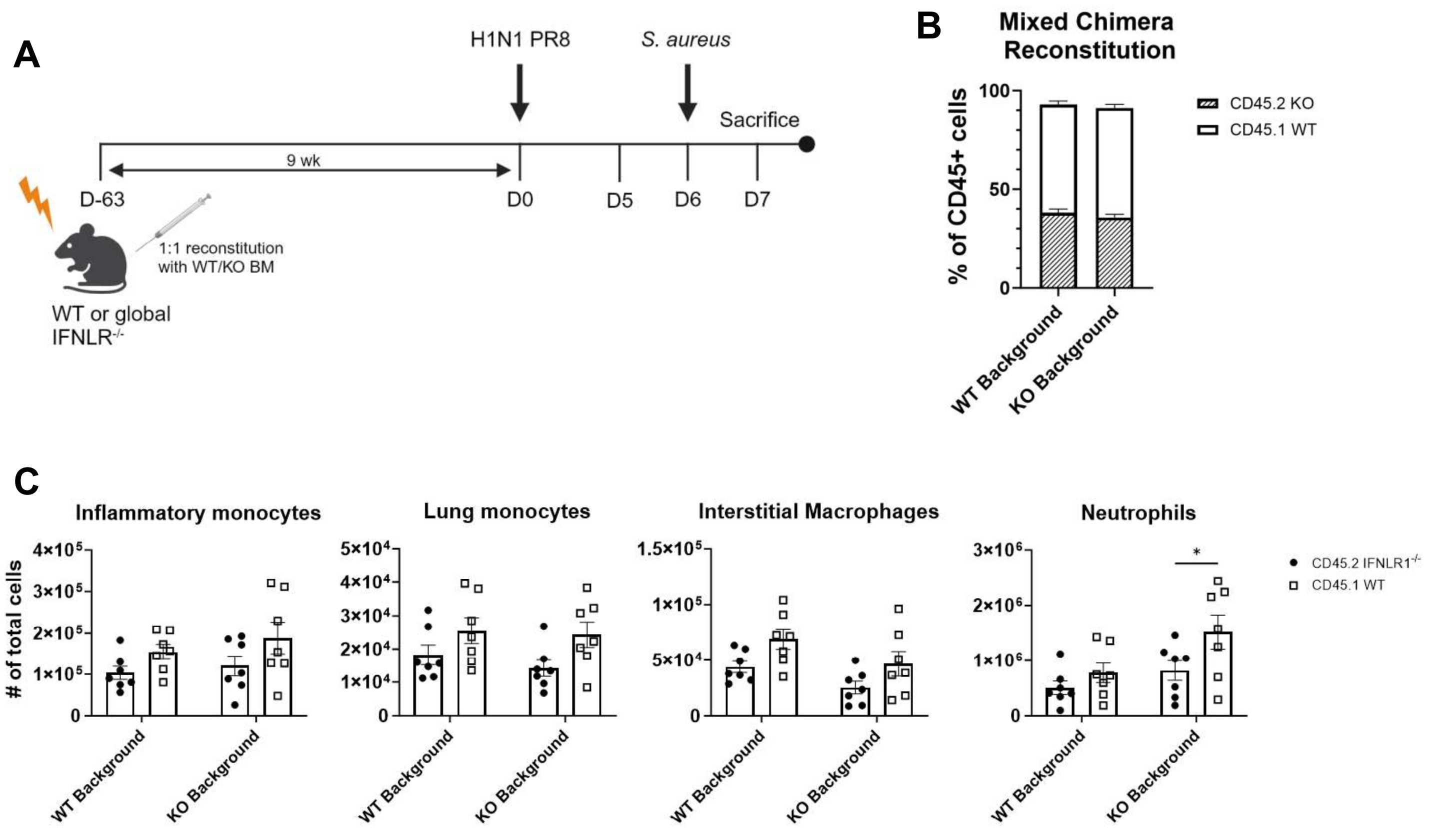

S5 Figure. Mixed bone marrow chimeric reconstitution of WT and IFNLR1$^{-/-}$ hosts. A. WT or IFNLR1$^{-/-}$ mice were sublethally irradiated and reconstituted with 1:1 WT:IFNLR1$^{-/-}$ bone marrow for 9 weeks before being super-infected. Figure created on Biorender.com. B. Reconstitution of bone marrow cells was comparable between WT and IFNLR1$^{-/-}$ cells in both mouse backgrounds. C. Total counts of immune cell recruitment to the lung was not significantly altered by host genotype or immune cell genotype, with the exception of neutrophils in IFNLR1$^{-/-}$ hosts (host: KO n=8, WT n=8). p values: *<0.05, **<0.01, ***<0.001, ****<0.0001
