# Supplementary material for: Cell-intrinsic regulation of phagocyte function by interferon lambda during pulmonary viral, bacterial super-infection
Source: PLoS Pathog. 2024 Aug 23;20(8):e1012498. doi: 10.1371/journal.ppat.1012498 (PMC11376568; doi:10.1371/journal.ppat.1012498)
Supplement: S6 Fig — A. S100A8-Cre-IFNLR1f l/f l mice show no difference in influenza m transcript levels when infected with influenza alone compared to Cre- controls (n = 6). B. Weight loss between S100A8-Cre- IFNLR1f l/f l and Cre- controls is unchanged during single influenza infection (n = 6). C. Weight loss between S100A8-Cre-IFNLR1f l/f l and Cre- controls is unchanged during single 24 hour MRSA infection (cKO n = 7, Cre- n = 6). D. Production of type I IFNs (ifnb) is not altered between S100A8-Cre-IFNLR1f l/f l mice and Cre- controls during super-infection, influenza, or MRSA infection. (PDF) [file ppat.1012498.s006.pdf]

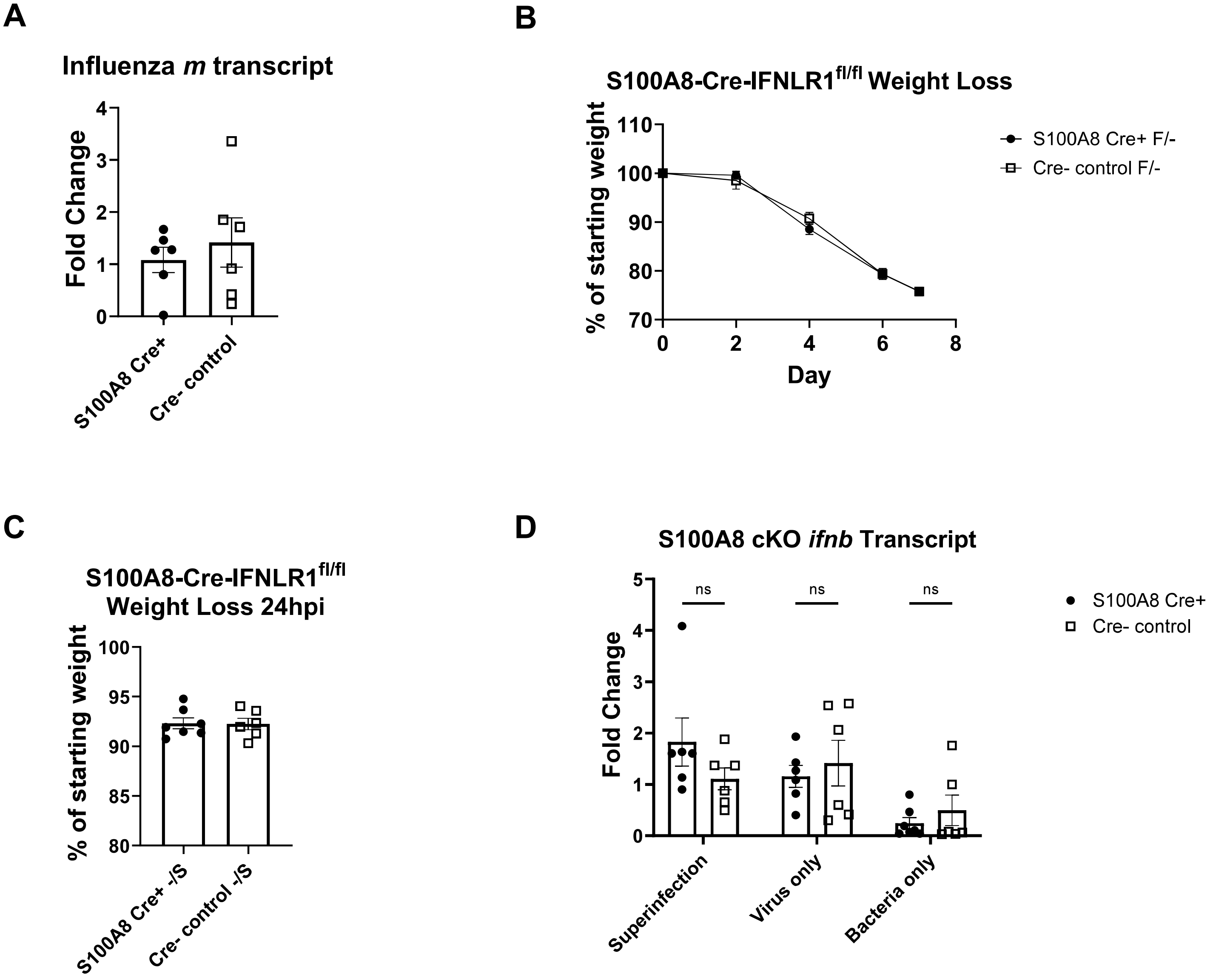

S6 Figure. Influenza controls show no difference in S100A8-Cre-IFNLR1$^{fl/fl}$ mice compared to controls. A. S100A8-Cre-IFNLR1$^{fl/fl}$ mice show no difference in influenza *m* transcript levels when infected with influenza alone compared to Cre- controls (n=6). B. Weight loss between S100A8-Cre-IFNLR1$^{fl/fl}$ and Cre- controls is unchanged during single influenza infection (n=6). C. Weight loss between S100A8-Cre-IFNLR1$^{fl/fl}$ and Cre- controls is unchanged during single 24 hour MRSA infection (cKO n=7, Cre- n=6). D. Production of type I IFNs (*ifnb*) is not altered between S100A8-Cre-IFNLR1$^{fl/fl}$ mice and Cre- controls during super-infection, influenza, or MRSA infection.
