# Supplementary material for: Cell-intrinsic regulation of phagocyte function by interferon lambda during pulmonary viral, bacterial super-infection
Source: PLoS Pathog. 2024 Aug 23;20(8):e1012498. doi: 10.1371/journal.ppat.1012498 (PMC11376568; doi:10.1371/journal.ppat.1012498)
Supplement: S7 Fig — A. CX3CR1 Cre specificity was tested in Cre+ mice (cKO) after Tamoxifen injections through detection of an eYFP reporter by flow cytometry (cKO n = 5, Cre- n = 6). B. ifnlr1 transcripts were measured by qPCR in tamoxifen-treated and–untreated mice (treated n = 4, untreated n = 4) C. Influenza m transcript levels were shown to be significantly reduced in CX3CR1-Cre-IFNLR1f l/f l mice compared to Cre-controls during single influenza infection. D. CX3CR1-Cre-IFNLR1f l/f l mice or Cre- controls were infected with influenza alone and show comparable weight loss over infection time-course (n = 5). E. Production of type I IFNs (ifnb) is not altered between CX3CR1-Cre-IFNLR1f l/f l mice and Cre- controls during single influenza infection (n = 5). F. Transcripts of sftpc, scgb1a1, and tjp1 were measured in lungs of cKO and control mice 24 hours after S. aureus infection (cKO n = 8, Cre- n = 8). All data is from 2 replicates. p values: *<0.05, **<0.01, ***<0.001, ****<0.0001. (PDF) [file ppat.1012498.s007.pdf]

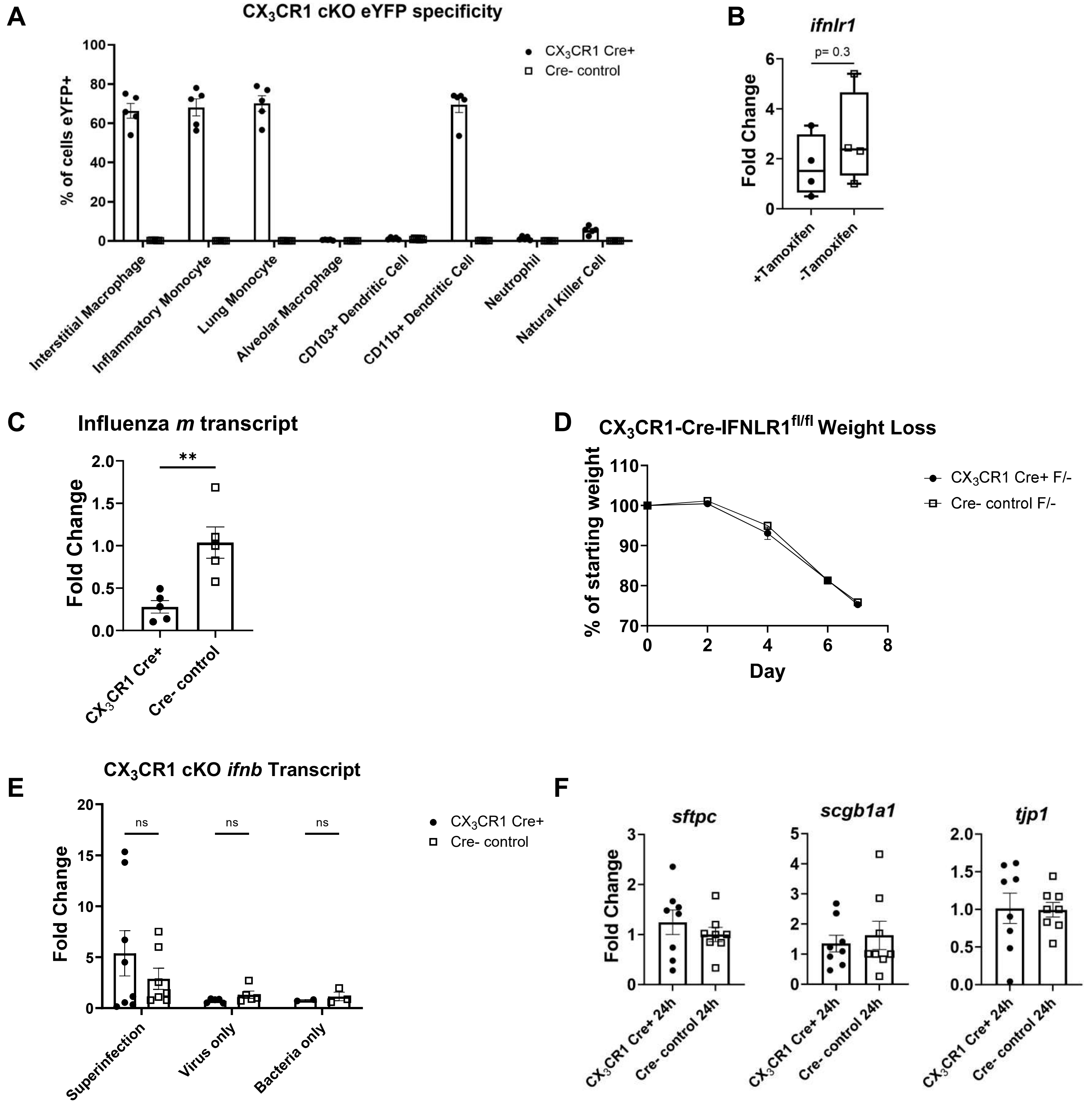

S7 Figure. CX_3CR1-specific depletion of IFNLR1 does not largely impact endpoints after super-infection, lowers viral burden. A. CX_3CR1 Cre specificity was tested in Cre+ mice (cKO) after Tamoxifen injections through detection of an eYFP reporter by flow cytometry (cKO n=5, Cre- n=6). B. *ifnlr1* transcripts were measured by qPCR in tamoxifen-treated and –untreated mice (treated n=4, untreated n=4) C. Influenza *m* transcript levels were shown to be significantly reduced in CX_3CR1-Cre-IFNLR1$^{fl/fl}$ mice compared to Cre- controls during single influenza infection. D. CX_3CR1-Cre-IFNLR1$^{fl/fl}$ mice or Cre- controls were infected with influenza alone and show comparable weight loss over infection time-course (n=5). E. Production of type I IFNs (*ifnb*) is not altered between CX_3CR1-Cre-IFNLR1$^{fl/fl}$ mice and Cre- controls during single influenza infection (n=5). F. Transcripts of *sftpc, scgb1a1,* and *tjp1* were measured in lungs of cKO and control mice 24 hours after *S. aureus* infection (cKO n=8, Cre- n=8). All data is from 2 replicates. p values: *<0.05, **<0.01, ***<0.001, ****<0.0001
