# Supplementary material for: Cell-intrinsic regulation of phagocyte function by interferon lambda during pulmonary viral, bacterial super-infection
Source: PLoS Pathog. 2024 Aug 23;20(8):e1012498. doi: 10.1371/journal.ppat.1012498 (PMC11376568; doi:10.1371/journal.ppat.1012498)
Supplement: S8 Fig — A. Frequencies of dsRed+ phagocytes was not altered between cKO mice and Cre- controls at 6h (open bars) or 12h (hashed bars) after bacterial infection (6h open bars: cKO n = 7, Cre- n = 7; 12h hashed bars: cKO n = 6, Cre- n = 5). B. Bacterial burden at earlier timepoints of 6h (left) or 12h (right) show no difference between CX3CR1-Cre-IFNLR1fl/fl mice and Cre- controls (6h open bars: cKO n = 7, Cre- n = 7; 12h hashed bars: cKO n = 6, Cre- n = 5). All data is from two replicates. p values: *<0.05, **<0.01, ***<0.001, ****<0.0001. (PDF) [file ppat.1012498.s008.pdf]

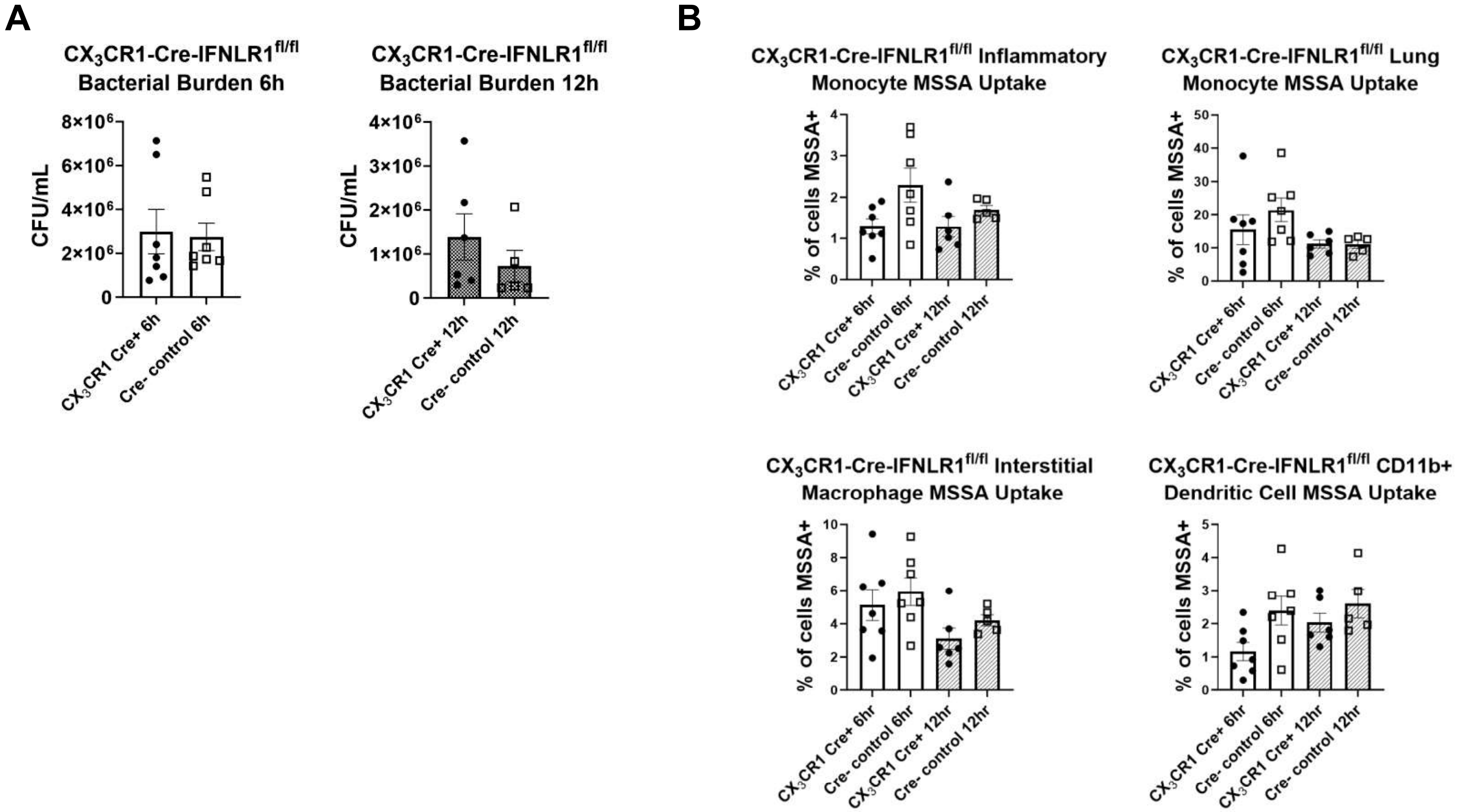

S8 Figure. CX$_3$CR1-specific depletion of IFNLR1 does not impact bacterial uptake A. Frequencies of dsRed+ phagocytes was not altered between cKO mice and Cre- controls at 6h (open bars) or 12h (hashed bars) after bacterial infection (6h open bars: cKO n=7, Cre- n=7; 12h hashed bars: cKO n=6, Cre- n=5). B. Bacterial burden at earlier timepoints of 6h (left) or 12h (right) show no difference between CX$_3$CR1-Cre-IFNLR1$^{fl/fl}$ mice and Cre- controls (6h open bars: cKO n=7, Cre- n=7; 12h hashed bars: cKO n=6, Cre- n=5). All data is from two replicates. p values: *<0.05, **<0.01, ***<0.001, ****<0.0001
