# Supplementary material for: Cell-intrinsic regulation of phagocyte function by interferon lambda during pulmonary viral, bacterial super-infection
Source: PLoS Pathog. 2024 Aug 23;20(8):e1012498. doi: 10.1371/journal.ppat.1012498 (PMC11376568; doi:10.1371/journal.ppat.1012498)
Supplement: S9 Fig — Flow cytometry gating for immune cell populations was performed as outlined. (PDF) [file ppat.1012498.s009.pdf]

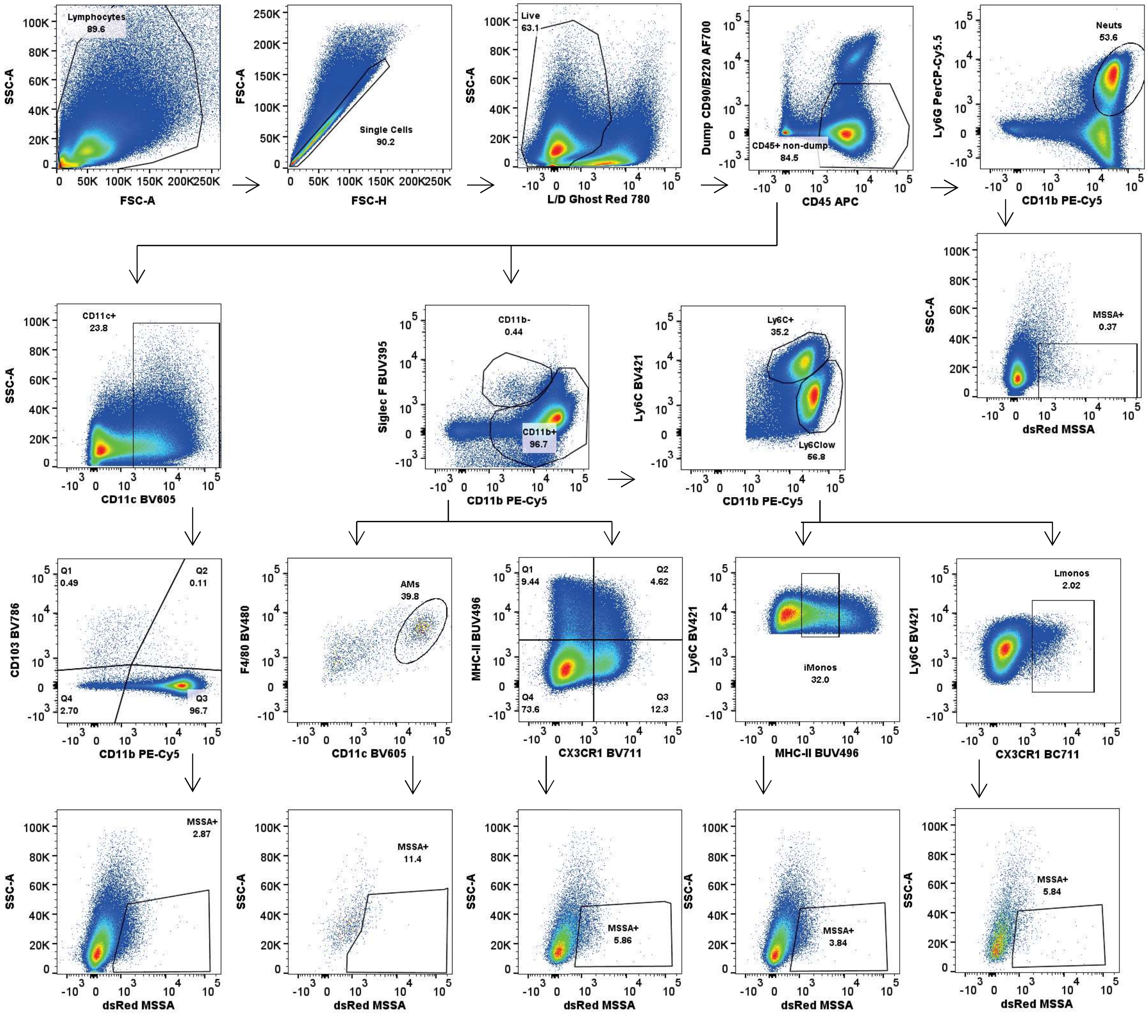

S9 Figure. Gating strategy for myeloid cell subsets. Flow cytometry gating for immune cell populations was performed as outlined.
