# Supplementary material for: Cell-intrinsic regulation of phagocyte function by interferon lambda during pulmonary viral, bacterial super-infection
Source: PLoS Pathog. 2024 Aug 23;20(8):e1012498. doi: 10.1371/journal.ppat.1012498 (PMC11376568; doi:10.1371/journal.ppat.1012498)
Supplement: Attachment — Submitted filename: Response to Reviewers 5-15-24.docx [file ppat.1012498.s012.docx]

Reviewer #1: Major

Comment 1 (C1): For all experiments mice are all infected with 900 PFU PR8, but what this represents in terms of lethality on its own is not included in the manuscript. For example, in Figure 1, IAV only controls are still losing weight at the conclusion of the experiment and it is unclear whether superinfection impacts mortality. IAV-only controls are also critical for interpretation of Figures 5 and 6 where the cell types targeted are known to be important for IAV infection on its own.

Response 1 (R1): We agree with the importance of IAV-only controls. Our lab has previously shown that 900 PFU of PR8 is not lethal to wild type B6 mice through day 21 of infection (Huckestein et al, Am J Path 2023), which we have added as a reference into the manuscript. We have also added IAV-only controls for the conditional S100A8 KO and CX_3_CR1 KO models as requested (S6A-B Fig & S7C-D Fig). We find that there is no difference in weight loss between experimental mice and their control counterparts. There is no change in viral burden in S100A8 KO mice, however conditional CX_3_CR1 KO mice have significant reductions in IAV matrix protein transcript compared to control mice at the time of super-infection.

C2: The S100A8-Cre mice are not included in the methods section. However, it seems, according to Jax, that these are the common name for Mrp8-Cre mice known not to be neutrophil specific. This critical point should be clarified.

R2: We thank the reviewer for pointing out this oversight and have added details of this mouse line into the Methods section. Additionally, we have added references that outline cell types targeted by this S100A8 (MRP8)-Cre line, mainly neutrophils with low off-target effects seen in several myeloid populations (Abram et al, 2014). While this Cre is not 100% specific to neutrophils, we do not see any phenotype in our model, supporting the conclusion that targeting IFNLR1 on neutrophils lacks an effect and have added this discussion point at Page 24 Line 466.

C3: In addition, the disconnect between enhanced MSSA uptake in neutrophils in the global KO and no difference in the outcome of Figure 5 should be discussed in greater detail

R3: We have addressed this in the Discussion section of the manuscript, starting on Page 23 Line 436.

C4: In general, the manuscript is under-cited. There are many publications that have investigated the role of IFN-lambda in regulating IAV pathogenesis and describing the role of IFN-lambda in immune cell functions (even in the context of IAV infection) that are not included or discussed. Importantly, how these defects during IAV-only potentially contribute to the defects observed during bacterial superinfection are not discussed.

R4: We agree with the reviewer and have added a number of new references that strengthen experimental rationale and discussion points. New references include numbers 5-7, 10-19, 29, 43-46, 55, 57, 59-61, and 64-78.

Reviewer #1: Minor

C1: Fig1F, I have no info to interpret the statistics. What was compared?

R1: We have included specifics on these statistical tests in each Figure legend, Page 53 Line 1015. Comparisons were run between IFNLR1^-/-^ and WT mice within each infection type, and significance is shown on the IFNLR1^-/-^ section of the heatmap.

C2: Thp1 cell experiments are not convincing at all, differences between 0.9 and 1 % if phagocytosing cells, despite having statistical significance, are not biologically relevant (see Fig 7C). do Thp1 cells even respond to IFN-λ? Did they measure?

R2: Based on suggestions given by other reviewers, we have elected to remove THP-1 data from the manuscript in order to dedicate more time to a relevant human cell model in the future. However, for informational purposes differentiated THP-1 cells do express IFNLR1 and produce ISGs upon stimulation.

C3: Overall the experiments, sample size and statistics are not described in a sufficiently clear manner.

R3: We appreciate this comment and have confirmed that sample sizes were included for all experiments, located in the Figure Legends. Statistical testing has been elaborated upon when unclear, such as in the heatmap found in Figure 1. We have also elaborated on the models used throughout the Discussion and hope that this clarifies experimental design choices.

Reviewer #2: Major

Comment 1 (C1): I do not understand what the authors claim in figure 2. Slight differences in IL17 and Il22 levels are detected, Il-17 delivery has similar effects in WT and IFNLR1 ko (in an experiment in which WT and KO do not differ in bacterial burden) and blockade of CXCL16 (unclear why it was chosen) doesn’t affect bacterial burden. The involvement of the communication between IFN-λ and IL-17 pathway in this process is unclear.

Response 1 (R1): We agree that the CXCL16 blockade was not sufficiently compelling to link IFNλ and IL-17 production during super-infection and have removed these data. In our experience with adenoviral vector treatments, the control adenovirus can alter the course of infection; we agree the lack of significance between Ad-eGFP KO and WT bacterial burden is misleading from the conclusions of Figure 1 and are confident that this is due to the effects of the physical adenovirus administration. In order to strengthen the link between IFNλ and IL-17, we have exchanged the Ad-IL-17 experiment in the previous Figure 2B with a converse experiment where we blocked IL-17A with a neutralizing antibody in IFNLR1 KO mice. This experiment showed that neutralization of IL-17A in super-infected IFNLR1^-/-^ mice worsened bacterial burden compared to isotype control mice, with concurrent decreases in neutrophil recruitment to the lung. We have also discussed the link between IFNλ and IL-17 in more detail within the Discussion section, starting on Page 20 Line 383.

C2: In figure 3 ( and throughout) the important control of uninfected mice is missing, especially for the FACS plots. How did thea author chose where to place the threshold for phagocytosis vs no phagocytosis? Additionally, it doesn’t seem like a population is picking up dsRED MRSA while another is not, but rather that there’s a global shift in the whole sample. Is it because everyonbe is phagocytosing a bacterium with low fluorescence? Are there any cell types (i.e. T cells) that don’t phagocytose to show as control? Most puzzling is that while the authors here claim that upto 90% of the cells phagocytose mrsa at 24h, in the very next figure, the authors show a phagocytosis rate of 7% at best.

R2: We thank the reviewer for this comment and suggestion of using uninfected mice and non-phagocytic cell populations to ensure that gates are drawn appropriately. dsRed+ gates have been re-drawn according to the edge of the population seen in CD90+ non-phagocytic cells (in both global IFNLR1 KO and WT mice), and these reference gates are now located in the supplemental data (S3 Fig). Additionally, we have also double-checked gates using a vector control bacterium that had an empty plasmid transfected in. These newly drawn gates have reduced the percentage of dsRed+ cells to be more consistent with later models. However, we do also think that the incomplete IFNLR1 deletion seen in our conditional KO mouse lines may also reduce the impact on phagocyte activity during super-infection, resulting in more modest frequencies of bacteria+ cells.

We also agree that the dsRed+ population in super-infected KO mice seems to be a continuous increase rather than a discrete population as seen in mice infected with pHrodo-red labeled MRSA. We understand that this is a point of concern, but have several hypotheses for the difference in staining patterns. Firstly, the RN6390 MSSA strain that we used was transfected with a plasmid containing the dsRed gene, meaning that all bacteria possess and produce the dsRed protein. Conversely, a culture of USA300 MRSA is labeled with pHrodo red after overnight growth, and it is possible that not 100% of the bacteria in the culture are successfully labeled. These baseline differences between fluorescent labels could explain the different types of positive populations. Secondly, the goal of assessing dsRed+ populations is to determine the overall uptake by a phagocyte, while the goal of assessing pHrodo red+ populations is to quantify the portion of phagocytes that possess bacteria within an acidic phagolysosome. Because the dsRed+ populations in the KO and WT mice are simply quantifying the frequency of cells that have any internalized bacteria within any type of phagosome, rather than identifying a specific environment within the cell at a specific timepoint, a more continuous increase in dsRed+ cells is to be expected. Even given this reasoning, however, we have performed super-infection experiments in global IFNLR KO and WT mice with pHrodo-red labeled MRSA 24 hours post infection, as suggested, to directly compare to our dsRed-MSSA data. We see that both experiments trend together, with increased frequencies bacteria+ phagocytes in global KO mice (S4 Fig), and, while this new pHrodo red data is not significant at 24 hours, it is also consistent both with data in CX_3_CR1 Cre+ mice at 6h and 24h post bacterial infection and with the notion that pHrodo red is capable of detecting early steps of bacterial clearance, which would occur at earlier timepoints after infection, likely at 4 or 6 hours post bacterial infection. We have also discussed the differences between dsRed and pHrodo red models in the Discussion, starting on Page 27 Line 526.

C3: Imagestream analyses are important in this context but why did the author not show a FACS-like dot plot from this experiment? And why did they only show 4 positive cells?

R3: We have added in additional images from these ImageStream experiments. Four cells were initially chosen and shown as representative images of all positive cells based on advice from our flow cytometry core, but we understand that only showing four cells may be misleading as to the number of dsRed+ cells.

C4: In figure 4 Along with the problem of the vastly different % between experiments the differences between WT and KO cells are modest an not always statistically significant.

R4: We agree that differences between WT and IFNLR1 KO cells specifically in global IFNLR1 KO host mice are modest, and we think that this is likely due to crosstalk between epithelial cells and immune cells during super-infection. We have elaborated on the model in the Discussion section starting on Page 23 Line 436, but we hypothesize that in WT mice, IFNλ is able to act on phagocytic cells both directly and indirectly. Our mixed chimeric mice support this hypothesis, as frequencies of dsRed+ cells in both WT and KO cells from KO host mice are modestly increased when compared to WT and KO cells from WT host mice; we intend to study this further in the future to provide more insights into this crosstalk. The inclusion of neutrophils in Figures 4C-D was chosen specifically given previous data showing a strong influence of IFNλ on this cell type, and we found the lack of a direct effect on neutrophils in this model to be very interesting based on previous published literature, leading into the use of the conditional S100A8 KO mice in Figure 5.

C5: In figure 6, as the most important readout is phagocytosis, why didn’t they show facs plots? Where are the statistical analyses of fig. 6E? What is the significance of the IDO expression? Is the effect dependent on phagocytosis or epithelial repair?

R5: We have added in representative flow plots and statistics into Figure 6E. We also completely understand the questions regarding IDO. While IDO has been shown to both be regulated by IFNλ with negative impacts for super-infection and expressed by several immune populations, we have elected to remove this piece of data in agreement with reviewers’ comments and confusion. We think that this removal increases clarity of Figure 6 and may be interested in re-visiting IDO more fully in the future.

C6: Finally in figure 7 the authors show a plot in which phagocytosing cells and non-phagocytosing cells are clearly separated. Why didn’t the author use the same model throughout? (pHrodo labeled bacteria?). While the data seem more solid ( despite only trends in certain populations) it is unclear whether the analyzed cells respond to IFN-λ at all. In fact, the RNAseq data don’t seem to support a cell intrinsic role for IFN-λ. For starters, among the differentially expressed genes, IFNLR1 doesn’t figure. The genes differentially expressed and above the pval threshold are very few, their fold change is very low and I could not identify canonical ISGs. Moreover, the genes in the heatmap are not the same than the volcano plot, how were they identified?

R6: We appreciate the reviewer’s comment and suggestion about using pHrodo red-labeled bacteria in earlier models and have used this idea to strengthen the uptake data presented in Figure 3. We agree that it is troubling that IFNLR1 is not in the list of DEGs between treated and untreated mice. Although we present qPCR data for IFNLR1 in the supplemental data (S7B Fig) which shows a trend towards a decrease in expression in tamoxifen treated mice. We have also performed additional analyses to specifically identify genes within the interferome that are significantly different between treated and untreated mice (Fig 8C). We have replaced the initial heatmap with genes specifically within the interferome for increased relevance and to draw more meaningful conclusions.

Reviewer #2: Minor

C1: It would be useful to determine viral copy number prior to superinfection on day 6 to determine the defect in IAV-only controls.

R1: We have added data from influenza-only conditional knockout controls, including weight loss and viral matrix protein transcripts (S6A-B Fig & S7C-D Fig). Interestingly, we found that CX_3_CR1-Cre-IFNLR1^fl/fl^ mice have significantly reduced matrix protein transcripts compared to Cre- controls (S7C), but no difference in S100A8-Cre-IFNLR1^fl/fl^ mice versus controls (S6A).

C2: Why IL-17 is investigated when there are no significant differences observed in Figure 1 is not discussed

R2: The Lincoplex kit used in this study seems to be relatively insensitive in regard to IL-17 levels, as our laboratory has had poor results with this specific cytokine and kit in all of our infection and mouse models. Additionally, the data in Figure 1 is derived from lung homogenate where IL-17 signal is quite low. We have previously shown that type I IFNs inhibit type 17 immunity during super-infection (Kudva et al, 2011), and other groups have linked IFNλ and IL-17 production in a variety of other models. Because of these previous data, we decided to run a more sensitive IL-17 ELISA on bronchoalveolar lavage samples, which did show a significant increase in super-infected IFNLR1^-/-^ mice compared to WT (Fig 2A).

Reviewer #3: Major:

Comment 1 (C1): Bacterial infection model: The authors state that they infect with 5 x 10e7 CFUs of Staph, of which only a fraction is recovered at 6 h (assuming a volume of roughly 1 ml of homogenate), with a further roughly 100 fold reduction between 6 and 24 h (Fig. 1B). Do these bacteria proliferate at all in the mice or would applying heat-inactivated bacteria give the same picture? While the difficulties with bacterial infection models are well appreciated, the numbers here do not suggest that Staph infects these mice particularly well, limiting the robustness of the infection model.

Response 1 (R1): The reviewer brings up an excellent point regarding the infectivity of mice with USA300 MRSA. While USA300 can infect mice and replicate, these bacteria have a much higher preference for human hemoglobin than mouse hemoglobin, which has been established for many years in the field. Because of this, mice are much less susceptible to USA300 infection and the bacteria are not able to release iron from mouse hemoglobin as efficiently. That being said, MRSA is still able to replicate to some level in murine models, and the hemoglobin preference of USA300 represents a known caveat to mouse studies in comparison to humans (Pishchany et al, 2010; PMID: 21147468). Additionally, bacterial burden in this study was enumerated using one small (upper right) lung lobe as opposed to the entire murine lung, meaning that these figures only represent a portion of the bacteria present in the whole lung at these timepoints. Our laboratory has found that mice infected with MRSA alone have an average of 1.5 x 10^5^ CFU/mL in their whole lung 24 hours after infection, which will be approximately 5- 10 fold higher in a super-infected mice at this time point.

C2: In 1C, it appears as if bacterial superinfection protects the wt mice from barrier damage vs single viral infection – how can this be explained? None of the known superinfection models seem to show such a protective effect. Also, wt mice are worse off than IFNLR KO in single viral infection – how come? While redundancy between IFN systems suggest that there may be no dramatic difference between wt and IFNLR KOs, this result is not in line with published data. For data in Fig. 1E, F it is not indicated when these measurements were done, and more generally, to make statements about inflammation, time course analysis of these data is needed. In addition, the bacterial load differences that are the key difference between wt and KO mice as stated by the authors already disappear in the control groups in Fig. 2B: here, the empty vector controls show no significant difference between wt and KO.
Overall, the base line infection data here leave important doubts about the model, its robustness and its predictability.

R2: We absolutely agree with the astute observation given by the reviewer. We repeated IAV-only control experiments in global IFNLR1 KO and WT mice to confirm the results from Figure 1C, and now see no significant differences in airway protein concentration between KO and WT mice. This set of bars in Figure 1C now shows a high and low cluster in the IAV-only WT group, and we hypothesize that the initial experiment shown previously could have had one of several physiological variations, likely from housing conditions or health prior to IAV infection. We hypothesize that airway protein levels are comparable between super- and IAV-infected mice due to the timepoint at which samples were collected, but this is an interesting observation that may require kinetic study in the future. We have added collection timepoints into the figure legend for Figure 1E and have generated a new supplemental figure containing many of the analyses performed in Fig 1 at 6 hours post bacterial infection rather than 24 to allow for better comparisons over multiple timepoints (S1 Fig).

C3: Link to type 17 responses: Fig. 2A, when is this measured? The authors need to keep in mind that bacterial burden drops very rapidly and that cytokine changes may be very dynamic, so this is difficult to determine by looking at just one time point. As pointed out already above, in Fig. 2B, the base line difference between wt and KO should be preserved in the GFP vector – treated groups but this is not the case. Furthermore, the only significant difference here is in the KOs +/- additional IL-17 - but these are the mice that already have more IL-17, while wt mice with lower IL-17 levels to start with should have a stronger effect from IL-17 addition, which is not the case – this set of data does not support much the authors’ argument. In addition, compared to the massive differences in CFUs found in other superinfection settings, the differences here are subtle , and it is difficult to understand how much biological significance there is in these small differences.

R3: To this reviewer’s point throughout, we have clarified collection timepoints throughout the manuscript. The reviewer also brings up an excellent point about Ad-eGFP controls losing significance between global IFNLR1 KO and WT mice, and we have exchanged the Ad-IL-17 experiment in the previous Figure 2B with a similar opposing experiment where we blocked IL-17A with a neutralizing antibody. This experiment showed that neutralization of IL-17A in super-infected IFNLR1^-/-^ mice worsened bacterial burden compared to isotype control mice, with concurrent decreases in neutrophil recruitment to the lung. In our experience with adenoviral vector treatments, control adenovirus alters the course of infection; we agree the lack of significance between Ad-eGFP KO and WT bacterial burden is misleading from the conclusions of Figure 1 and are confident that this is due to the effects of the physical adenovirus administration. Although it’s true that the differences between KO and WT mice are, while significantly different, subtle, it is unlikely that bacterial burden during super-infection will ever be ameliorated completely, thus making these small differences worth studying. Our laboratory has also previously shown that even a two-fold bacterial challenge difference results in significantly altered mortality in mice (Kudva et al., 2011).

C4: Fig. 2D,E: CXCL16 should BLOCK IL17/22 to prove the authors’ point, however the authors show only CFU data, not IL-17 protein, and il22 only by RNA. All this data is required to support the points being made here. MIP3a is not modifiec experimentally, why?

R4: We completely agree that the CXCL16 blockade presented was incomplete data linking IFNλ and IL-17 production during super-infection and have removed these data.

C5: Link to phagocytosis: In fig.3, how are cell subsets identified / pre-gated? The data shown here indicates that there is an overall shift in fluorescence, not two populations – how come? This looks like a 100:0 result which does not make much sense. In addition, the BM chimeras in fig.4 show Staph labelling that is very low – none of the high percentages of the full KO are seen here – how is this possible? The pHrodo data gives two clear-cut populations, this should be equally the case with the plain labelled bacteria. All this does not induce much confidence in this reviewer that the bacterial loading data is reproducible or interpretable.

R5: Based on this and another reviewer’s suggestion, we have gone back and re-gated dsRed+ cells using a CD90+ non-phagocytic population as a reference gate (now included in a supplemental figure, S3A Fig). We also agree that the dsRed+ population in super-infected KO mice seems to be a continuous increase rather than a discrete population as seen in mice infected with pHrodo-red labeled MRSA. We understand that this is a point of concern, but have several hypotheses for the difference in staining patterns. Firstly, the RN6390 MSSA strain that we used was transfected with a plasmid containing the dsRed gene, meaning that all bacteria possess and produce the dsRed protein. Conversely, a culture of USA300 MRSA is labeled with pHrodo red after overnight growth, and it is possible that not 100% of the bacteria in the culture are successfully labeled. These baseline differences between fluorescent labels could explain the different types of positive populations. Secondly, the goal of assessing dsRed+ populations is to determine the overall uptake by a phagocyte, while the goal of assessing pHrodo red+ populations is to quantify the portion of phagocytes that possess bacteria within an acidic phagolysosome. Because the dsRed+ populations in the KO and WT mice are simply quantifying the frequency of cells that have any internalized bacteria within any type of phagosome, rather than identifying a specific environment within the cell at a specific timepoint, a more continuous increase in dsRed+ cells is to be expected. Even given this reasoning, however, we have performed super-infection experiments in global IFNLR KO and WT mice with pHrodo-red labeled MRSA 24 hours post infection, as suggested, to directly compare to our dsRed-MSSA data. We see that both experiments trend together, with increased frequencies bacteria+ phagocytes in global KO mice (S4 Fig), and, while this new pHrodo red data is not significant at 24 hours, it is also consistent both with data in CX_3_CR1 Cre+ mice at 6h and 24h post bacterial infection and with the notion that pHrodo red is capable of detecting early steps of bacterial clearance, which would occur at earlier timepoints after infection, likely at 4 or 6 hours post bacterial infection. . We have also discussed the differences between dsRed and pHrodo red models in the Discussion, starting on Page 27 Line 526.

C6: Within the experiment shown in fig.4, the differences may be statistically significant, but they are very very small - how do we know they actually have biological meaning? In addition, it is unclear to this referee why would this (small) difference in BM-derived cells would disappear in the KO recipient background. Overall these data are inconclusive as to the role of phagocytosis downstream on IFNL.

R6: We agree that differences between WT and IFNLR1 KO cells specifically in global IFNLR1 KO host mice are modest, and we think that this is likely due to crosstalk between epithelial cells and immune cells during super-infection. We have elaborated on the model in the Discussion section, starting on Page 23 Line 436, but we hypothesize that in WT mice, IFNλ is able to act on phagocytic cells both directly and indirectly. Our mixed chimeric mice support this hypothesis, as frequencies of dsRed+ cells in both WT and KO cells from KO host mice are modestly increased when compared to WT and KO cells from WT host mice; we intend to study this further in the future to provide more insights into this crosstalk. We appreciate that the absolute differences between WT and KO cells in the WT host are subtle, but the tight clustering of data points and statistical significance support the conclusion that this is biologically relevant, especially as CX_3_CR1-Cre-IFNLR1^fl/fl^ mice show protection against lethal super-infection compared to Cre- controls.

C7: This lack of conclusiveness is further compounded when differences in phagocytic capacities are not reproduces in myeloid-specific IFNL KO mice (Fig.6E): At this point, everything that the authors suggest up to here essentially collapses, and other, unrelated explanations are put forward, such as increased IDO production in wt vs. KO mice: this comes a bit out of the blue here and is completely unlinked to all prior data. In contrast, the increased bacterial burden (Fig. 6B) in CX3CR1 Cre KOs phenocopies full KOs which is fine, but why then do the mixed BM chimeras not show the same? Overall, three different models (full KO, BM chimeras with KO BM, myeloid cell-specific conditional KO) give three different outcomes, and the overlap is too partial to draw a conclusive picture of what is happening here.

R7: This comment is fair for the reviewer to make. While IDO has been shown to both be regulated by IFNλ with negative impacts for super-infection and expressed by several immune populations that have been studied in this manuscript, we have elected to remove this piece of data in agreement with reviewers’ comments and confusion. We think that this removal increases clarity of Figure 6 and may be interested in re-visiting IDO more fully in the future. We have re-worked several areas of the Results and Discussion sections to clarify and better explain the rationale and findings in each model. Regarding the bacterial burden, significant reductions in super-infected CX_3_CR1 cKO mice compared to Cre- controls supports the conclusion that IFNλ signaling on myeloid cells decreases activity during super-infection, like what is seen in global IFNLR1 KO. We hypothesize that differences in bacterial burden are lost in mixed chimeric mice because the introduction of KO immune cells (that are more effective at taking up bacteria) into an otherwise WT mouse is sufficient to normalize burden levels to what is seen in KO host mice, and this hypothesis has also been supported by the generation of standard bone marrow chimeras not included in this manuscript. We believe that crosstalk between epithelial cells and immune cells is incredibly important during super-infection, and that IFNλ is able to signal to immune cells both directly, to cells that express IFNLR1, and indirectly, via molecules that are induced by IFNλ signaling in airway epithelial cells. This is supported both by our results in the mixed chimeric mice, as explained previously, and in the CX­_3_CR1 cKO mice – differences in uptake and burden are more modest (and in some places, not significantly different) in super-infected cKO mice due to the ability of their stromal cells to still respond to IFNλ signaling. We appreciate this comment and the opportunity to explain nuances of the model and system more fully, starting on Page 23 Line 436 of the Discussion, continuing through Page 26 Line 505.

C8: Have the authors checked type I IFN levels in the IFNRL KOs, reduced type I IFN could explain most findings here…
Have the authors checked IFNLR levels on all the cells you are talking about here?

R8: We agree and have checked IFNβ levels in all models and have found no difference, we’ve added these data into the supplemental figures to strengthen conclusions (S6D Fig, S7E Fig). The comment regarding IFNLR1 expression brings up an excellent point, and identifying murine IFNLR1-expressing cells is quite difficult. Murine IFNLR1 antibodies and reagents are notoriously poor, but we have isolated CD11c+ cells from the lungs of naïve WT and global IFNLR1^-/-^ mice and found that these WT cells express *ifnlr1* transcripts (S2C Fig), which is absent in KO mice.

C9: In fig. 7, authors should do these experiment with BALF-derived MPh, no need to use an artificial model such as THP-1 cells. If they want to include human data they should use blood monocytes and MPh derived from them.

R9: We have weighed the pros and cons of these data and have elected to remove the THP-1 data from the manuscript. We agree with the reviewer that the use of a cell line is not comparable to animal models and think that pursuing a primary human cell model more fully in the future is a worthwhile endeavor to linking impacts of IFNλ in mice and humans during infection.

C10: Fig.8: Tamoxifen has clear effects on immune cells, so another key control that needs to be included is +TAM in Cre-neg cells. Fig.8A: why no unsupervised clustering for treatment samples?

R10: We have added unsupervised clustering into Figure 8. We absolutely agree that Tamoxifen-treated Cre- mice are an important control, but this represents a significant limitation in our approach. Because we sorted out all eYFP+ cells, which are only present in Cre+ mice, we were unable to sort out cells from Cre- mice in an equivalent measure. We also found that the CX_3_CR1 Cre did not target 100% of target cells (S7A Fig), meaning that using CX_3_CR1 to sort out cells would result in a small population of CX_3_CR1+ cells that should be Cre+ and are not, complicating interpretation of any results found. We have discussed these limitations in more detail in the Discussion section, starting on Page 26 Line 507, taking advantage of existing literature regarding the kinetics and effects of Tamoxifen in mice. Studies have shown that the effects of tamoxifen only occur during the washout period, after which the animal returns to baseline, and these immune-related impacts are seen at higher doses than what we gave in this study, largely 3 mg and above. Additionally, all other experiments performed in CX_3_CR1-Cre-IFNLR1^fl/fl^ mice have included appropriate Tamoxifen-treated Cre- controls.

Reviewer #3: Minor:

C1:Line 106 fix this phrase

R1: We have fixed this phrasing and appreciate this note, now found on Page 7 Line 110.

C2: Line 130 rephrase, IFNL does NOT mediate bact clearance

R2: We have modified this line, now found on Page 9 Line 140.

C3: Line 332 no you do not show this – it does not happen in the myeloid- or NPh-specific IFNLR KOs.

R3: We have altered this line for clarity, now found on Page 20, Line 380.

C4: Fig. 6C: p=0.2 is not an indication of significant differences, this would happen by chance in one of 5 attempts…

R4: We agree that a p value of 0.2 is not impressive and have repeated this experiment. The survival difference between CX_3_CR1-Cre-IFNLR1^fl/fl^ mice and Cre- controls in Figure 6C now reaches significance.
