# Supplementary material for: Cell-intrinsic regulation of phagocyte function by interferon lambda during pulmonary viral, bacterial super-infection
Source: PLoS Pathog. 2024 Aug 23;20(8):e1012498. doi: 10.1371/journal.ppat.1012498 (PMC11376568; doi:10.1371/journal.ppat.1012498)
Supplement: Attachment — Submitted filename: Reviewer Response.docx [file ppat.1012498.s013.docx]

Reviewer #1: This manuscript has been significantly improved and the authors have sufficiently addressed all reviewer comments. GEO Accession is still not available for RNAseq data set and deposition in repository should be completed prior to/at the time of publication.

R1) All sequencing data has been uploaded to Gene Expression Omnibus (GSE268994). This statement has been added to the methods section as requested. We also added details regarding the use of IL-17 neutralizing antibody that was not present in the original methods section.
